# Multitechnique characterization of eco-corona formation on airborne nanoplastics

**DOI:** 10.1039/d5ra03254g

**Published:** 2025-08-28

**Authors:** Anna Placci, Marta Fadda, Irene Coralli, Junjie Wang, Andrea Zattoni, Anna Luisa Costa, Raquel Portela, Andrea Mario Giovannozzi, Daniele Fabbri, Dora Melucci, Stefano Giordani, Barbara Roda, Pierluigi Reschiglian, Simona Ortelli, Alessio Sacco, Valentina Marassi

**Affiliations:** a Department of Chemistry “G. Ciamician”, University of Bologna Via Piero Gobetti 83 40129 Bologna Italy valentina.marassi@unibo.it; b Quantum Metrology and Nanotechnology Department, National Institute of Metrological Research (INRiM) Strada Delle Cacce 91 10135 Torino Italy a.sacco@inrim.it; c Department of Chemistry “Giacomo Ciamician”, Technopole of Rimini, University of Bologna Via Dario Campana 71 47922 Rimini Italy; d ByFlow S.R.L Via Dell’Arcoveggio 74 40129 Bologna Italy; e CNR-ISSMC, National Research Council of Italy – Institute of Science, Technology and Sustainability for Ceramics Via Granarolo 64 48018 Faenza RA Italy simona.ortelli@issmc.cnr.it; f CSIC-Instituto de Catalisis y Petroleoquimica (ICP) C/ Marie Curie, 2 28049 Madrid Spain

## Abstract

The increasing presence of micro- and nanoplastics in natural environments raises concerns about their interactions with biological particles such as pollen, that may act as carriers but could also undergo subtle chemical or structural changes, potentially influencing their ecological role. At the same time, the analytical and technological approaches used to investigate nanoplastic pollution mechanism can themselves raise concerns regarding their greenness. In this interdisciplinary study, we explored the interactions between multifloral bee pollen and polyethylene terephthalate nanoparticles (NanoPET) under environmentally relevant conditions using a multimodal analytical strategy combining AF4 (Asymmetrical Flow Field-Flow Fractionation) multidetection, Pyrolysis-GC-MS (py-GC-MS), Field Emission Scanning Electron Microscopy (FESEM), and dielectrophoresis-Raman spectroscopy (DEP-Raman). This approach aims to clarify nanoplastics exposure profiles and the associated potential health risk, as well as to promote more sustainable laboratory workflows. Pollen and NanoPET were first characterized individually by AF4, FESEM, and DEP-Raman, which provided their size distributions, morphology, and characteristic spectral signatures. Py-GC-MS offered detailed molecular fingerprints, especially for bee pollen, which had not been previously analysed with this technique. To assess the interaction between pollen and NanoPET, mixed samples were analysed using a “profilomic” approach based on changes in AF4 fractograms, UV/Vis and Raman spectra. Two distinct interaction mechanisms have emerged: the formation of a corona of soluble pollen-derived macromolecules around NanoPET, and the coating of pollen grains by NanoPET particles, as confirmed by FESEM imaging. DEP-Raman further confirmed the presence of interactions by separating non-interacting NanoPET particles and revealing spectra that included characteristic peaks of both pollen and NanoPET. Py-GC-MS analysis of fractions collected from AF4 processing of mixed samples also confirmed the presence of characteristic ions deriving from both components. Together, these findings highlight the formation of hybrid bio-nano structures and suggest potential ecological implications. Moreover, they demonstrate how multidimensional, low-impact analytical workflow can offer detailed insight into nanoplastics behaviour in complex biological matrices, paving the way for greener and more comprehensive environmental nanotoxicology studies.

## Abstract 2

A profilomic approach used AF4, UV/Vis and Raman to investigate pollen–NanoPET hybrid bio-nano structures and eco-coronas. This green, multidimensional analytical workflow gave insight on nanoplastics behaviour in complex biological matrices.

## Graphical abstract

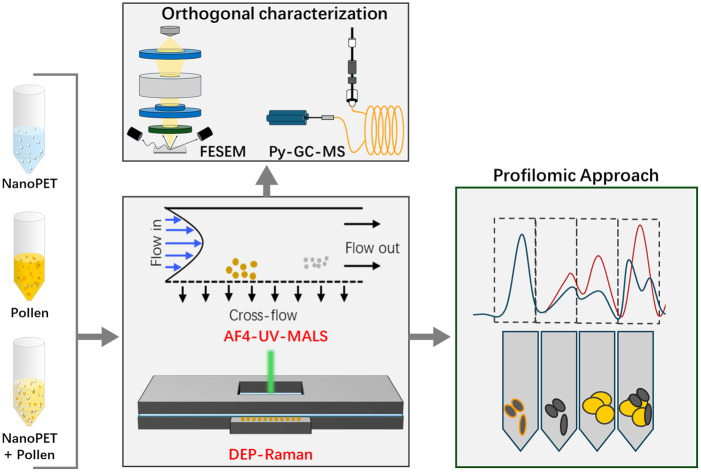

## 1 Introduction

Nanoplastics (NPs; length < 1 μm (ref. 1)) and microplastics (MPs; length between 1 μm and 5 mm^1^) are plastic particles, mainly obtained from the degradation of plastic residuals previously released in the environment due to anthropogenic activities.^2^ Their small size allows their transportation over long distances through their surroundings as well as cell permeability (especially NPs)^3^ and studies suggest their ubiquitous presence in the main environmental and biological matrixes (from water and air to food and blood).^4^ NPs and MPs can impact the ecosystem health acting both as vectors and carriers of pollutants in the environment, respectively by leaching out dangerous plastic additives and by absorbing pollutants due to their high surface area and binding affinity.^5,6^ Correlations between NPs/MPs and pathological conditions^2,7^ and ecosystem damage^8^ have been reported in literature. For example, concerning pollination, it has been demonstrated the dangerous role of the atmospheric NPs and MPs deposited by rains both on plants and on pollinators.^9^

Studies on the characterization and environmental behavior of such species are limited in the literature due to the overall difficulty of their characterization as well as to the interference of the involved matrixes; additionally, the smaller size of NPs makes certain analytical techniques inadequate for their accurate measurement, because of their insufficient spatial resolution or mass limits of detection.^4,10^ NPs and MPs change identity in the exposure media through the formation of a matrix dependent eco/bio-corona through the interaction and physi/chemisorption of available biomolecules and natural organic matter on their surfaces.^11,12^ To date, it remains largely unexplored how the corona determines NPs and MPs biological effects: therefore, assessing the nature of the corona as well as how such particles change due to its formation is of outmost importance.

Asymmetrical flow field flow fractionation (AF4), along with its miniaturized version hollow fiber flow field flow fractionation (HF5), is the most common and exploited field flow fractionation (FFF) variant.^13–15^ It provides a soft and native hydrodynamic size-based separation of species inside a hollow channel. Indeed, being a single-phase separation technique, the sample does not undergo stress during separation. Moreover, its operational flexibility allows the selection of a mobile phase that mimics the sample matrix, thus preserving its original state. Its wide separation range (1 nm to 20 μm) makes it suitable for the study of complex biological and environmental samples,.^16–18^ The coupling with diode array (DAD), differential refractive index (dRI), and multi angle light scattering (MALS) detectors allows a size, morphological, and spectroscopic characterization of the separated species as well as the observation of corona formation.^19,20^

Traditionally AF4-multidetection platforms have largely been endorsed as suitable technique for the analysis of nano plastics;^21^ however, the number of studies on complex matrices is reduced^22–24^ and focus only on detecting NPs in the matrix, without providing information on how the NPs interact with the matrix changing their nature and behavior. To address this methodological gap, we developed an approach based on an AF4 multidetection platform (DAD-MALS-dRI) to investigate the interaction of nanoparticles—using polyethylene terephthalate (PET) with a mean hydrodynamic diameter of 100 nm (referred to in this work as NanoPET) as model nanoplastic—and biological matrices, with pollen as example, in an aqueous environment. PET is one of the most abundant airborn nanoparticle found in urban and suburban areas.^25^ Bee pollen is a complex and varied multilayer structure mainly composed on average of carbohydrates (54%), proteins (21%), lipids (5%), fibers (9%) and other less abundant components^26^ which provide essential nutrients for bees and play a fundamental role in plant reproduction, critical for ecosystem health.^27^ Given its molecular diversity and compositional complexity, pollen was selected as a representative biological matrix to challenge the resolving power of our platform. The aim was to prove that relevant interaction patterns can be captured even in complex systems, without relying on component-specific or targeted analyses, which are often too demanding or impractical.

PET MPs and NPs have been detected ubiquitously across different environmental compartments, with concentrations varying widely depending on the matrix and proximity to pollution sources. In a remote high-altitude environment such as the Sonnblick Observatory, Kau *et al.* (2024) quantified PET micro- and nanoplastics in particulate matter, reporting average concentrations of approximately 35 ng m^−3^ in PM10 and 21 ng m^−3^ in PM1, with PET constituting about half of the total plastic content measured.^28^ These findings demonstrate that even remote mountain areas are subject to significant PET pollution. In urban and suburban regions of India PET MPs concentrations have been reported up to 158 ng m^−3^ in PM2.5, confirming the widespread presence and elevated levels of PET microplastics in air highly impacted by human activity.^29^ Furthermore, in terrestrial systems, the presence of PET MPs has also been documented in agricultural soils in Bangladesh, with concentrations ranging from 0.6 to 3.7 PET particles per gram of soil, indicating the persistence and penetration of PET into soil environments relevant for ecological processes and terrestrial life.^30^ PET micro- and nanoplastics, once introduced into complex matrices such as soil, water, and air, rapidly interact with a myriad of environmental biomolecules including proteins, polysaccharides, lipids, and humic substances. These interactions lead to the formation of an eco-corona that alters the surface chemistry and aggregation behavior of PET particles, influencing their environmental fate and toxicity. For example, studies have shown that soil metabolites can adsorb onto PET microplastics, modifying pollutant binding,^31^ while in aquatic systems, eco-corona formation on PET facilitates microbial colonization and toxin production.^32^ Despite these insights, specific investigations on interactions between corona-coated PET particles and pollen remain lacking, highlighting an important research gap. Understanding these interactions could unveil novel bio-nano hybrid mechanisms influential in ecosystem dynamics.

To the best of our knowledge, to date there are no studies on the interaction of pollen with MPs/NPs, despite the importance of this matrix in our ecosystem. However, it is reasonable to hypothesize that various components of pollen may interact with plastic nanoparticles, potentially leading to the formation of hybrid bio-nano structures. These interactions may involve adhesion, encapsulation, or mutual surface modification, depending on environmental conditions. Surface properties of PET nanoparticles, such as hydrophobicity and charge, as well as the biochemical features of the pollen constituents, are likely to influence the extent and nature of the interactions. Therefore, understanding these mechanisms is essential to assess how they may affect nanoparticle identity and behavior in biological systems.

With this high-throughput analytical technique, we aim to tackle the system's complexity, deepening the understanding of exposure profile, and to contribute to the development of effective methods for analyzing potential health risks from nano plastics. The developed approach allowed the characterization of the particle size change underwent by NanoPET in the presence of the water-soluble macromolecular pollen species and of the eco-corona formed around the particles. The proposed method is fast, cheap, and works in native and greener conditions, since neither sample preparation nor external surfactant or salt in the AF4 carrier composition, or organic solvents are required. The offline coupling with a pyrolysis–gas chromatography–mass spectrometry (Py-GC-MS) system and a field-emission scanning electron microscope (FESEM), as well as the employment of Raman microspectroscopy enabled by dielectrophoresis (DEP) on unfractionated samples, allowed to confirm the AF4 results as well as to obtain an unprecedented characterization of the soluble pollen species and NanoPET eco-corona composition. Overall, this work represents the first attempt with an AF4 system to assess how an aqueous pollen environment influences the exposure identity of airborne NPs. We believe that this detailed and information-rich approach will elucidate the impact of eco-corona on NPs behavior while also translating research and analytical approaches towards greener setups.

## 2 Experimental

Polyethylene terephthalate nanoparticles (NanoPET) dispersed in water at a concentration of 4.9 mg mL^−1^, spherical in shape with an average hydrodynamic diameter of 100 nm (*D*_50_) in intensity and 69.41 nm in Volume (*D*_v_) – as measured by dynamic light scattering (DLS) – supplied by CSIC through PlasticsFatE project (sample ID = PET_b001). In addition, the suppliers provided the *ζ*-potential and pH of the dispersed particles, whose values testify the good stability of the solutions. The properties of the plastic sample are shown in Table S1.

Polystyrene (PS) nanospheres with sizes of 50 nm (PS_50 nm), 200 nm (PS_200 nm), and 300 nm (PS_300 nm), dispersed in water at a solids content of 1% m/v, were purchased from Duke Scientific Corporation. Due to their high uniformity and stability, these nanoparticles were used as standards for AF4 method development and size calibration. Their properties are shown in Table S2.

Multifloral bee pollen was purchased from Alce Nero (Castel San Pietro Terme, Italy) and it came in the form of small, irregularly shaped pebbles of varying sizes and colours.

HPLC grade water (purchased from Merck) was used as mobile phase and for the preparation of all samples and standard solutions.

Sodium chloride (NaCl, Cat. No. S9888, ACS reagent, ≥99.0%), sodium phosphate dibasic dihydrate (Na_2_HPO_4_·2H_2_O, Cat. No. 30435, 98.5–101.0%) and potassium phosphate monobasic (KH_2_PO_4_, Cat. No. 795488, ACS reagent, ≥99.0%) were obtained from Sigma Aldrich; potassium chloride (KCl, Cat. No. 60130, ≥99.5%) was obtained by Fluka. These salts were used to prepare phosphate buffer saline (PBS) 1× solution (NaCl 8 g L^−1^, KCl 0.2 g L^−1^, Na_2_HPO_4_·2H_2_O 1.43 g L^−1^ and KH_2_PO_4_ 0.2 g L^−1^) employed as mobile phase and diluent solution to mimic a physiological environment.

### 2.1. NanoPET dilution samples for characterization in water and PBS 1×

In order to obtain homogeneous samples in native condition, the NanoPET dispersion was sonicated in a bath sonicator (35 kHz) for 1 min at room temperature before use and then diluted gravimetrically in ultrapure water to reach a concentration of 0.49 mg mL^−1^. The diluted suspension was sonicated for an additional 1 min before injection. The solution was used for a preliminary characterization of NanoPET and to verify the feasibility of the developed method. Two different mobile phases, PBS 1× and ultrapure water, were used to test the stability of the particles in different conditions.

### 2.2. Pollen solutions for characterization

The pollen suspended samples were prepared by mixing 50 ± 5% mg of pollen pebbles with 1 mL of ultrapure water or PBS 1×. The suspensions were soft vortexed for 3–5 min until all the pebbles were completely dissolved, forming a dark yellow turbid liquid that was centrifuged for 3 min at 302 RCF in an Eppendorf MiniSpin AG 22331 centrifuge (Hamburg, Germany) to remove all the insoluble matter and impurities, obtaining a clear yellow supernatant, representing our pollen sample. The pollen samples were then analyzed with an AF4 multidetection platform in their original concentration with a volume of 5 μL using both ultrapure water and PBS 1× as mobile phases, to understand the stability of pollen in different environments, respectively simulating real-life and physiological conditions.

### 2.3. Pollen and NanoPET mixtures

NanoPET–pollen mixture samples were prepared by keeping constant the pollen concentration (pollen solution diluted at a 1 : 4 ratio) while NanoPET content changed between 1.9 and 30.0 μg mL^−1^ (MIX1 to MIX5, Table S3). As pollen reference sample we considered the sample obtained by diluting 1 : 4 the of pollen suspension. Meanwhile, for the plastic references, several solutions were prepared containing only NanoPET at the same concentration as the mixtures. Table S3 reports the list of mixture samples along with concentrations of their components. It was observed that pollen was unstable and tended to precipitate and visibly degradate, so all the samples that contained pollen supernatant needed to be analysed within a few days after preparation.

### 2.4. AF4 analysis

AF4 analyses were performed with an AF2000 MultiFlow FFF system (Postnova Analytics GmbH, Landsberg am Lech, Germany) comprised of a solvent degasser PN7520, two isocratic pumps PN1130 used for solvent delivery and focus flow control, and a Flow FFF control module with integrated electronic interface and a cross-flow syringe pump (AF2000 Module) to generate the transversal flow inside the channel. The separation was performed using 10 kDa MW cut-off regenerated cellulose membranes (Postnova Analytics GmbH) in a 300 mm × 60 mm × 40 mm channel with 350 μm thick spacer. PS standards for method development and size calibration were diluted in ultrapure water before injection (PS_50 nm was diluted 1 : 200, PS_200 nm and PS_300 nm were diluted 1 : 400, reaching a final concentration of 0.05%, 0.0025%, and 0.0025% m/v, respectively). The PS suspensions were analysed with an injection volume set to 5 μL using ultrapure water as mobile phase. The selected PS standards (50, 200, and 300 nm) were chosen due to their well-defined, monodisperse size distributions, which make them ideal for calibration and method development in AF4. The chosen size range was guided by preliminary data on the dimensions of NanoPET and colloidal components present in the pollen supernatant, ensuring the method's ability to resolve all expected species. AF4 method development is described in the SI.

The AF4 separation method consists of three phases: focus-injection, elution, and rinse. In the focus-injection step, the injected sample is confined to a narrow band by two opposing mobile phase flows. This generates a transversal flow that pushes the particles from the upper wall towards the porous wall of the channel (accumulation wall). This causes the sample components to reach an equilibrium position across the channel thickness, balancing their diffusivity with the perpendicular force generated by the cross flow. During the elution phase, a longitudinal flow (detector flow) is maintained in the channel, and separation can be achieved through the application of a gradient of cross flow. During the rinse step, the cross flow is stopped, and the detector flow flushes out all the residuals from the channel. The setting parameters of the method are shown in Table S4: the focusing step was performed for 7 min, with a detector flow of 0.5 mL min^−1^, injection flow of 0.5 mL min^−1^ and a cross flow of 2 mL min^−1^. During the elution step the cross flow is set at 2 mL min^−1^ for 2 min, then exponentially decrease from 2 mL min^−1^ to 0.01 mL min^−1^ within 40 min (exponent 0.1). During the final stage, to ensure the complete elution of all the species, the cross flow was maintained to 0.01 mL min^−1^ for 10 min, followed by 5 min without cross flow. Method parameters were selected to achieve optimal separation resolution across the size range of interest, from NanoPET to larger pollen-derived colloidal species. The mobile phase was ultrapure water to mimic native conditions. On-line detection of analytes was carried out through a PN3242 UV-Vis/DAD detector (AF4-UV/Vis) and a PN3621 MALS detector (AF4-MALS). NovaFFF version 2.2.0.1 software was used to control the instruments, set separation parameters, collect and elaborate data. Mixture and reference samples were analysed both with the AF4-DAD-MALS platform. The AF4 volume of injection was 15 μL and the mixtures were injected both right after preparation and after overnight storage at room temperature to verify if incubation time could affect the formation of pollen-based eco-corona over NanoPET surface. Since the AF4 technique is non-destructive, it not only offers analytical potential but also serves as a preparative tool, enabling the collection of sample fractions for further offline analysis. In this study, fractions from reference and mixture samples were manually collected at the outlet during selected elution time windows and subsequently analyzed by pyrolysis-gas chromatography–mass spectrometry and field-emission scanning electron microscopy.

### 2.5. FESEM analysis

Morphological analysis on NanoPET particles dispersed in water, pollen and of NanoPET–pollen mixture samples from AF4 were carried out using a field emission scanning electron microscope (Supra 40, Zeiss, Germany). For FESEM analysis, specimens were prepared by dripping the fractions collected onto a silicon wafer fixed to an aluminum stub. The drops were evaporated at room temperature in the ambient atmosphere and gold metalized. The FESEM image of unprocessed NanoPET was analyzed with the ImageJ software (version 1.52p), calculating the diameter of at least 150 particles. The mean, the mode and the size distribution were calculated with the values obtained.

### 2.6. Py-GC-MS analysis

To perform Py-GC-MS analyses for chemical characterization, the sample volume must be reduced to a maximum of 80 μL and, mostly, water must be removed, being incompatible with GC-MS. Therefore, a specific set of samples was prepared by increasing the NanoPET concentration by 5×. Following AF4 separation, two fractions were collected in 2 mL Eppendorf tubes and they were lyophilized. 1,1,1,3,3,3-hexafluoro-2-propanol (HFP) was used to solubilize the samples, after testing its capability to solubilize both NanoPET and pollen. For the quantitative recovery of NanoPET the HFP solutions were posed under rotational shaking for 2 h at 4 rpm, then quantitatively transferred into the 80 μL-pyrolysis cup (Eco-cup LF, Frontier Lab), 40 μL at a time, gradually evaporating HFP under a gentle N_2_ flow, and finally the cup was heated on a plate at 60 °C for 5 min.

Analytical conditions were optimized by adding to the sample before analysis 5 μL of the derivatization reagent tetramethylammonium hydroxide (TMAH, 25 wt% in methanol, Sigma Aldrich) for thermally assisted hydrolysis and methylation (THM) as well as tri-*tert*-butylbenzene (TTB, 10 μL of 10 ppm solution in toluene) and tridecanoic acid (C13, 5 μL of 100 ppm solution in methanol) as internal standards (ISs) for quantification by gas chromatography. The choice of the two ISs was guided by the will to mimic both the aliphatic and aromatic portions of PET pyrolysis products (TTB) and the oxygenated compounds which undergo methylation (C13), also assessing the derivatization performance of the runs.

The Py-GC-MS analysis was carried out using a multi-shot pyrolizer (EGA/Py-3030D, Frontier Lab) online interfaced with a gas chromatograph and quadrupole mass spectrometer (7890B and 5977B, Agilent Technology). The samples were introduced into stainless steel pyrolysis cups which were individually released into the microfurnace of the pyrolyzer, at 600 °C in He inert atmosphere. Pyrolysis cup preparation was performed as follows: solid samples (*e.g.*, pollen pebbles or pollen supernatant) were weighted directly inside the pyrolysis cup, whereas liquid samples (*e.g.*, NanoPET or mixture solutions in HFP) were injected into the cup using a micro-syringe and the solvent was completely evaporated under gentle N_2_ flow followed by heating at 60 °C for 5 min. Then, quartz wool (pre-treated at 400 °C for 4 h) was added to the cup to improve the contact between the sample and the derivatization reagent. TMAH and IS solutions were finally added just before the run. The selected temperature was already used in literature to analyze microplastics from environmental samples. Evolved gas analysis (EGA) tests confirmed that the temperature was appropriate for the complete thermal degradation of pollen (data not shown). Once pyrolysis products were generated inside the furnace, they were transferred to the GC column (HP-5MS, stationary phase 5%-phenyl, 95%-methyl polysiloxane 30 m × 0.25 mm i.d. × 0.25 μm film thickness, Agilent Technology), thanks to a constant He flow (Py-interface and GC-inlet set at 280 °C, split ratio 20 : 1). For each run the oven started at 50 °C, held for 2 min, then temperature increased to 310 °C with a 7 °C min^−1^ ramp, held for 8 min. Mass spectra of pyrolysis products were recorded under 70 eV electron ionization in the *m*/*z* 35–600 range at 2.6 scan sec^−1^. The temperature of the ion source and the quadrupole were 230 °C and 150 °C, respectively.

### 2.7. DEP-Raman analysis

Pollen, and NanoPET/pollen MIX1-5 were chemically characterized by coupling dielectrophoresis (DEP) and Raman microspectroscopy in the experimental setup previously reported,^33^ consisting of an electrode quadrupole as the bottom of a chamber, a gasket, and a glass coverslip ceiling for optical microscopy. 5 μL sample volumes were injected at a time into the DEP device. The electrical field in the DEP cell was a sinusoidal voltage of 5 V peak to peak at a frequency of 1 MHz, resulting in negative dielectrophoresis and net forces on the samples directed towards the center of the cell, where the confocal volume of the Raman microscope was located. DEP is a phenomenon that depends on the electrical properties of the particles and the medium as well as on the dimensions of the particles, and this property was advantageously employed in this study. At this combination of electrical conditions, isolated NanoPET smaller than 100 nm in diameter were neither accumulated nor measured, as positive dielectrophoresis moved them away from the analyzed volume: only pollen particles, both bare and combined with NanoPET, were transported in the active area of the cell and their spectra measured. The pollen–NanoPET accumulation time before acquisition was 30 s. The spectra were acquired by different injections to sample the greatest amount of pollen. To acquire the characteristic spectrum of isolated NanoPET, the voltage frequency was increased to 20 MHz to achieve negative dielectrophoresis.

The spectra were collected using a 60× water immersion objective (1.1 numerical aperture) in a confocal Raman Imaging Microscope DXRxi (Thermo Scientific, US) using an excitation wavelength of 532 nm, a laser power of 20 mW and a spectrograph confocal pinhole aperture of 50 μm in diameter. Spectra were collected as an average of 60 scans with an exposure time of 1 s (1 min per spectrum), with 5 cm^−1^ average spectral resolution and a spectral range of 500–3100 cm^−1^.

## 3 Results and discussion

### 3.1. AF4 profiling and FESEM morphological characterization of reference pollen and NanoPET samples

Since we aimed to investigate bee pollen and NanoPET in natural and native environments, we screened water and PBS 1× solutions as explorative mobile phases in AF4.

**Fig. 1:**
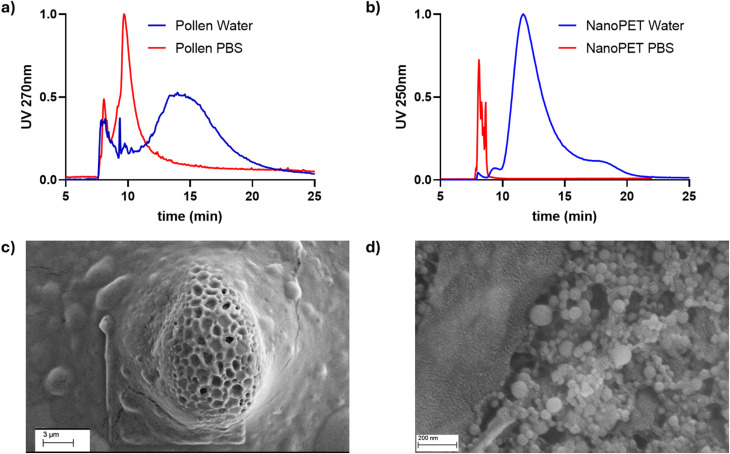
AF4-UV/Vis comparative normalized fractograms obtained in water and PBS 1× mobile phases of (a) pollen and (b) NanoPET sample. FESEM images of (c) pollen and (d) NanoPET sample.

Pollen grains generally reach 10–100 μm sizes together with the co-presence of nanometer-scale substructures^34^ and soluble molecules:^35^ to operate in suspension and liquid carriers, focusing onto nano-molecule and nano–nano interactions, we therefore selected the soluble portion of pollen. This was done *via* precipitation and removal of the high-micron species. When pollen samples are injected using PBS 1× as mobile phase, as shown in Fig. 1a, the pollen elutes out at the beginning of the elution step, not completely separating from the void peak. We hypothesize that the interplay between the membrane and the pollen is influenced by the ionic strength of the mobile phase, resulting in a repulsive interaction that leads to weak retention of the sample. The NanoPET sample, that carries negative charge (*ζ* = −31 ± 1 mV, Table S2), cannot elute out of the channel smoothly under the same physiological conditions (Fig. 1b), likely because of a strong attractive interaction between the plastic and the membrane. However, both samples can be eluted easily using water as mobile phase (Fig. 1a and b, blue profiles). Therefore, the latter was chosen as both mixing medium and mobile phase. This choice also constituted a representative medium for the interaction between pollen and plastic nanoparticles in the natural environment. Although environmental factors such as humidity and temperature can influence the extent and nature of interactions between the NanoPET and biological matrices, including them would significantly increase the system's complexity. Therefore, in this initial study, we focused on simplified and controlled aqueous conditions to validate the analytical platform and approach.

FESEM analysis of pollen supernatant (Fig. 1c) revealed micrometric grains (∼15 μm) with oblong-ellipsoid shape and a nanometric surface ornamentation. Larger grains were not detected, as they were removed during the centrifugation steps in the sample preparation process. FESEM imaging of unprocessed NanoPET (Fig. 1d) shows the presence of spherical particles with a relatively wide diameter distribution. The image analysis on more than 150 particles revealed that the mean diameter was 50 ± 17 nm, with a mode of 55 nm confirming the width of the particle size distribution observed in Fig. 1d. NanoPET particle size distribution obtained by FESEM image analysis on more than 150 particles is shown in Fig. S1.

**Fig. 2:**
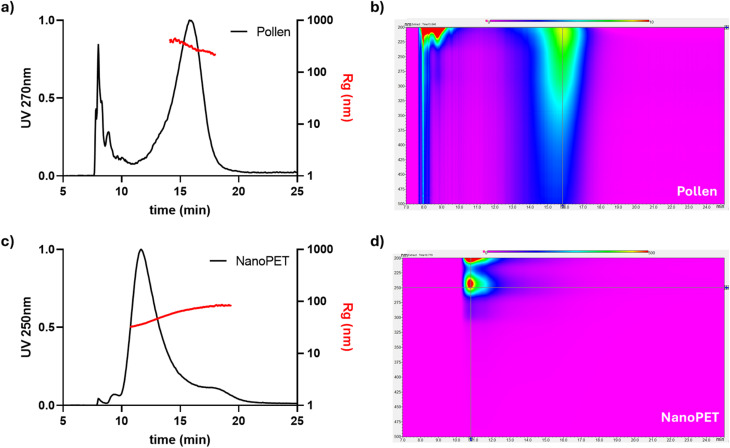
For pollen (top) and NanoPET (bottom): On the left, overlay of AF4-UV/Vis normalized fractograms at 270 nm for pollen (a) and at 250 nm for NanoPET (c) with relative distribution of gyration radius (*R*_g_). On the right, representative absorption spectra (iso-absorbance plot, (b) for pollen and (d) for NanoPET).

Pollen and NanoPET individual suspensions were characterized *via* the developed AF4 method, in water, and with UV and MALS detection (Fig. 2a and c). Pollen showed a first peak at lower retention time (*R*_t_: 8 min) and a second broad band with a larger size range (*R*_t_: 16 min, gyration radius distribution: 220–420 nm, Fig. 2a), both with a peculiar absorption profile (isoabsorbance plot, Fig. 2b). Absorbance spectra of those two populations are shown in Fig. S2.

As shown in Fig. 2c, the main peak of NanoPET has a retention time of 11.5 min showing a gyration radius (*R*_g_) distribution ranging from 30 to 85 nm, highlighting a partial aggregation of the NanoPET that emphasizes the importance of MALS analysis to accurately define the size distribution. Fig. 2d shows the characteristic absorption maximum of PET at 250 and 290 nm.

In the developed method, pollen and NanoPET peaks have different retention times, UV/Vis spectra, and size distributions: this scenario is very promising for the use of the AF4 multidetection platform as a fast tool for NanoPET recognition in such a complex matrix. Indeed, a simple variation of the iso-absorbance plot, without the need for additional and more time-consuming chemical characterization, allows to monitor NanoPET in pollen in a “profilomic” based approach. To better visualize the system in a two-dimensional output, we focused on the signals at 490 nm, typical for pollen (or rather exclusive to pollen), and at 250 nm, corresponding to PET absorption maximum and the most sensitive wavelength for this component. In this way, perturbation in the mixtures at the two wavelengths with respect to the profile recorded for unmixed samples (reference samples) can directly show the presence of NanoPET and its interactions with pollen, which can then be confirmed by more specific chemical characterization (*e.g.*, Py-GC-MS, Raman spectroscopy). This approach offers a streamlined procedure to focus on the most informative parts of the profile —where significant deviations from the reference samples are observed—thus allowing immediate detection of matrix modifications.

### 3.2. Pyrolysis-GC-MS characterization of reference pollen and NanoPET samples composition

Py-GC-MS was used to chemically characterize NanoPET, pollen and pollen supernatant, with the aim to identify pyrolysis products, and markers for qualitative and quantitative purposes. Thanks to its interest and well-defined chemical composition, markers of PET by unreactive pyrolysis and in the presence of TMAH are well known,^36^ rendering the analysis of NanoPET *via* Py-GC-MS is relatively simple. Unreactive pyrolysis of PET generates many aromatic compounds with carboxyl (*e.g.*, benzoic acid or (vinyloxycarbonyl)benzoic acid) or ester groups (*e.g.*, vinyl benzoate, divinyl terephthalate, than-1,2-diyldibenzoate) reflecting the monomers of PET synthesis, ethylene glycol and terephthalic acid.^36^ When PET is pyrolyzed in a mixture with other materials (*e.g.*, other polymers or matrices), carboxylic acids can be involved in secondary reactions because of their high reactivity,^37^ possibly biasing PET identification and quantification. Conversely, reactive pyrolysis with TMAH of PET generates a single dominant pyrolysis product, namely dimethyl terephthalate,^36^ minimizing the possibility of secondary reactions and increasing the analytical sensitivity.

**Fig. 3:**
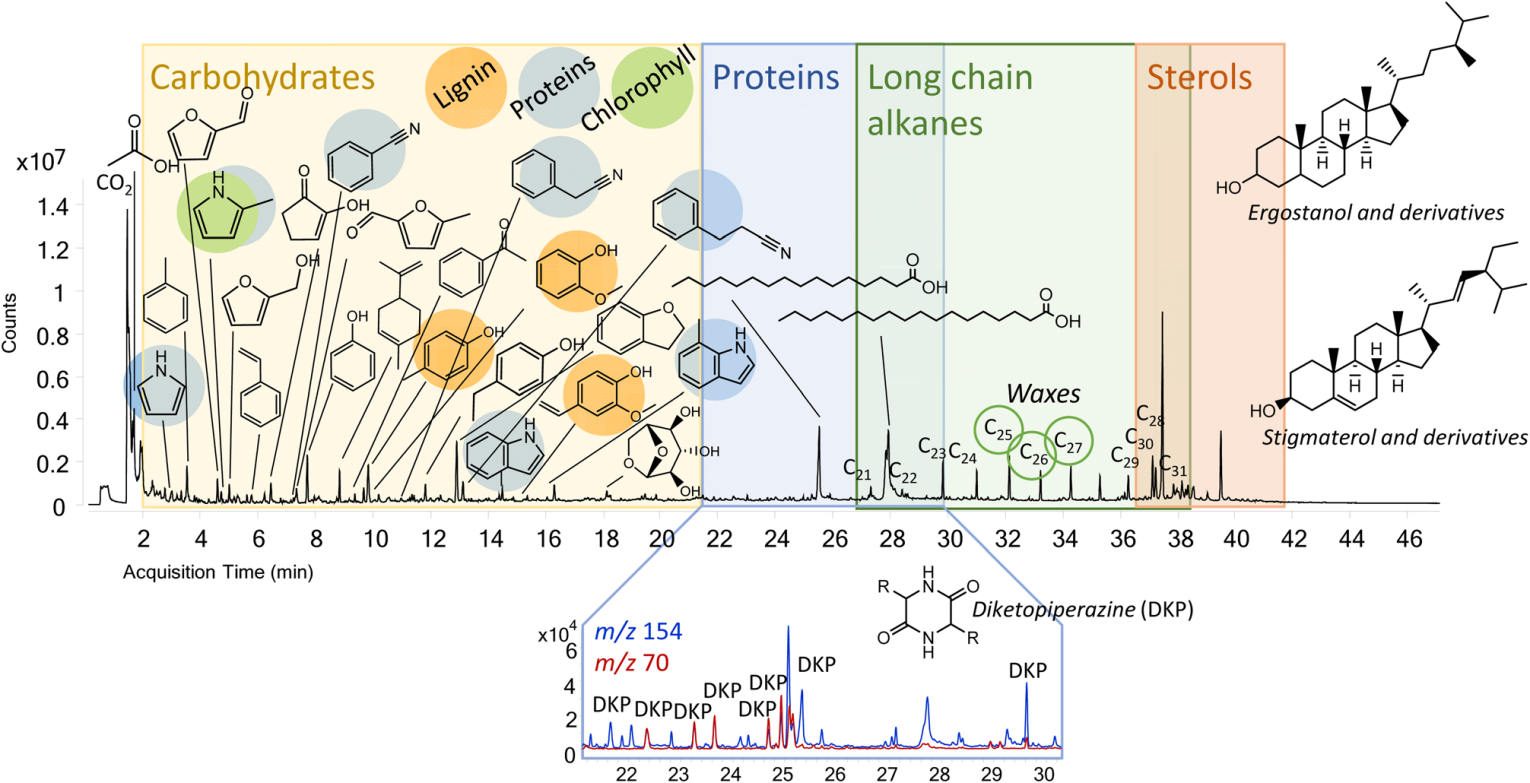
Pyrogram of 0.15 mg of pollen pebbles.

In contrast to NanoPET, the Py-GC-MS data of bee pollen, a biological matrix with a very complex chemical composition, are more difficult to interpret and, as far as we know, there are no published studies on the analytical pyrolysis of bee pollen. Pyrolysis products of pollen have been investigated in paleontology as proxies to obtain past information of Earth, that is stored in organic remains.^38^ According to Rodriguez-Polit *et al.*, the chemical composition of heterofloral bee pollen is highly variable because of the variety of the involved plants and the presence of nectar and bee saliva. However, proteins, carbohydrates, lipids, amino acids, phenolic compounds, and fibers are the main components.^39^ Levoglucosan and furfurals present the pyrogram of bee pollen pebbles (Fig. 3) confirm the presence of carbohydrates in the sample, whereas the phenols and hydroxyphenols are typical of the pyrolysis of polysaccharides or lignocellulosic biomass. In addition, guayacol, creosol and vinyl guaiacol suggest the presence of lignin 40, but the absence of syringol and its derivatives indicates that those molecules may also be pyrolysis products of some natural hydroxycinnamic acids present in the sample. Nitrogen-containing compounds, such as pyrrole, benzonitrile, indole and diketopiperazines, were also found as indicators of the protein moiety.^41^ Among diketopiperazines, a marker of gluten was found in the diketopiperazine of proline and glutamic acid. Alkylpyrroles were also identified, but they are most probably related to the tetrapyrrole ring of chlorophyll.^42^ Finally, lipids are important components of pollen, as evidenced by the long chain *n*-alkanes detected from 21 to 31 carbon atoms with an odd to even preference typical of waxes, long chain fatty acids and sterols (*e.g.*, ergostanol, stigmasterol and their derivatives).

**Fig. 4:**
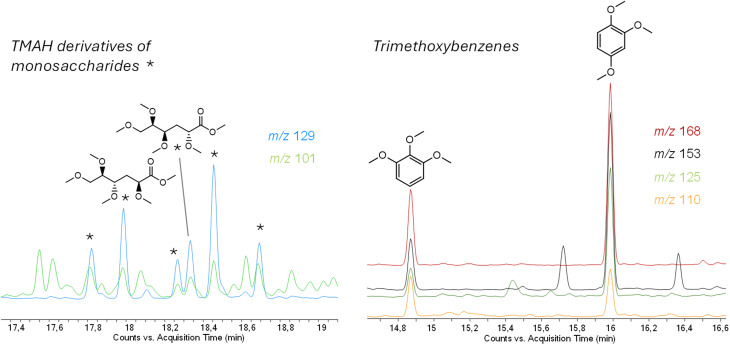
Extracted Ion Chromatogram (EIC) of pollen supernatant reactive pyrolysis with TMAH showing target ions of TMAH derivatives of monosaccharides (*m*/*z* 129, 101) and trimethoxybenzenes (*m*/*z* 168, 153, 125 and 110).

The pyrolysate composition of the supernatant pollen changed drastically. Lipid markers were fairly detectable, while markers of carbohydrates became predominant. Hydroxyphenyls guaiacyls and protein markers were revealed, but with a different pattern in comparison to original pollen; for instance, diketopiperazines were absent. TMH-TMAH pyrolysis confirmed the families of compounds detected by unreactive pyrolysis. Among carbohydrates, some monosaccharides were found, such as glucose and galactose, due to the presence of epimers of tetra-*O*-methyl-3-deoxy-hexonic acid methyl ester (d-arabino and d-ribo configuration) and tetra-*O*-methyl-3-deoxy-hexonic acid methyl ester (d-xylo and d-lyxo configurations) (Fig. 4, left).^43^ Moreover, 1,2,3-trimethoxybenzene and 1,2,4-trimethoxybenzene were detected (Fig. 4, right). According to the literature, their presence is usually related to lignin and cellulose, respectively.^43^

However, according to unreactive pyrolysis products, together with the fact that they were found both in pollen pebbles and supernatant pyrograms, they may state the presence of lignin or TMAH derivative pyrolysis products of natural hydroxycinnamic acids. Methyl-*p*-methoxycinnamate and methyl cinnamate assessed the presence of *p*-coumaric and cinnamic acids, which were not detected in unreactive pyrograms. Cinnamic acid is a precursor for lignin and flavonoids in higher plants and, according to Watson *et al.* 2012, coumaric acid and its derivatives are supposed to be monomers of the large bio-polymer of sporopollenin, which constitute the external wall of pollen grains.^44^ Moreover, ferulic acid (typical of wheat) and sinapic acid (widespread in fruits and cereals) were detected in their methylated structures, even though in very low amounts.^45,46^ Unreactive pyrolysis showed the complete loss of lipids in pollen supernatant, but in TMAH reactive pyrolysis methyl ester of some fatty acids were found. According to these findings, carbohydrates and hydroxycinnamic acids are the most distinctive categories of compounds in pollen supernatant, *i.e.* the matrix used in the interaction studies, and therefore the related molecules (under THM-TMAH conditions) may be used as proxies. Nevertheless, the use of TMAH requires careful consideration. The selection of appropriate experimental conditions is crucial to avoid incomplete derivatization especially for sterically hindered groups^47^ as well as loosely reactive hydrogens, such as those found in amides.^48,49^ In the context of this study, methylation of pyrolysis products of NanoPET resulted complete as well as those from pollen. IS C13 was used to assess the derivatization performance and, additionally, no free acids or alcohols were detected. Moreover, it was demonstrated that even in a complex organic matrix, the use of TMAH enhanced the detection of PET in comparison with unreactive Py-GC-MS.^50^ In general, the advantages of TMAH-Py-GC-MS for the simultaneous identification and quantification of polymers in complex matrices have been widely demonstrated for the reduction of secondary reactions, and the increase of both sensitivity and accuracy of its quantification.^37,48,51,52^ However, potential matrix interferences remain for polymers not affected by THM-TMAH, such as polystyrene and poly(vinyl chloride). Finally, competing reactions between NanoPET and pollen were not observed as they individually generated different patterns of methylated pyrolysis products that did not change upon TMAH-Py-GC-MS of NanoPET–pollen mixture.

These findings support the necessity of TMAH-assisted pyrolysis for achieving reliable analytical outcomes for NanoPET in pollen, underlining its essential role in overcoming the limitations of conventional Py-GC-MS in complex matrices.

### 3.3. Characterization of the interaction between NanoPET and pollen

#### 3.3.1. AF4 and FESEM analyses

**Fig. 5:**
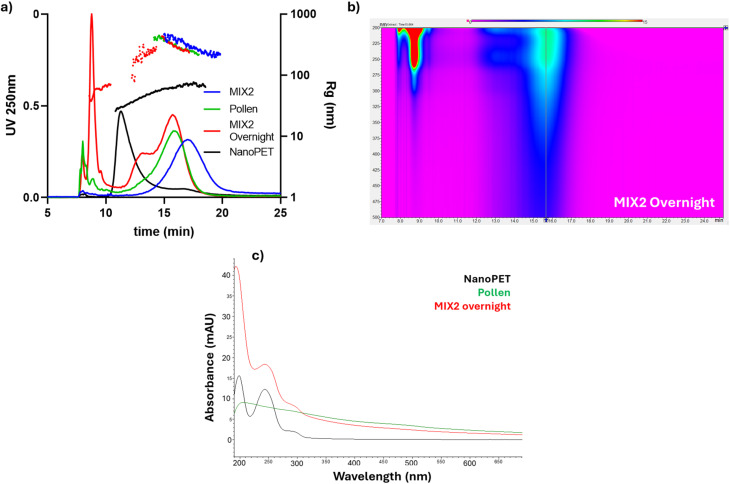
(a) Overlay of 250 nm fractograms of pollen reference, NanoPET 0.015 mg mL^−1^, MIX2, and MIX2_overnight samples and their corresponding *R*_g_ distributions; (b) UV/Vis two-dimension iso-absorbance plot image of MIX2_overnight (c) overlay of representative UV/Vis absorbance spectra of pollen (green profile), NanoPET (black profile) and the three population found in MIX2_overnight fractogram (red profile).

**Fig. 6:**
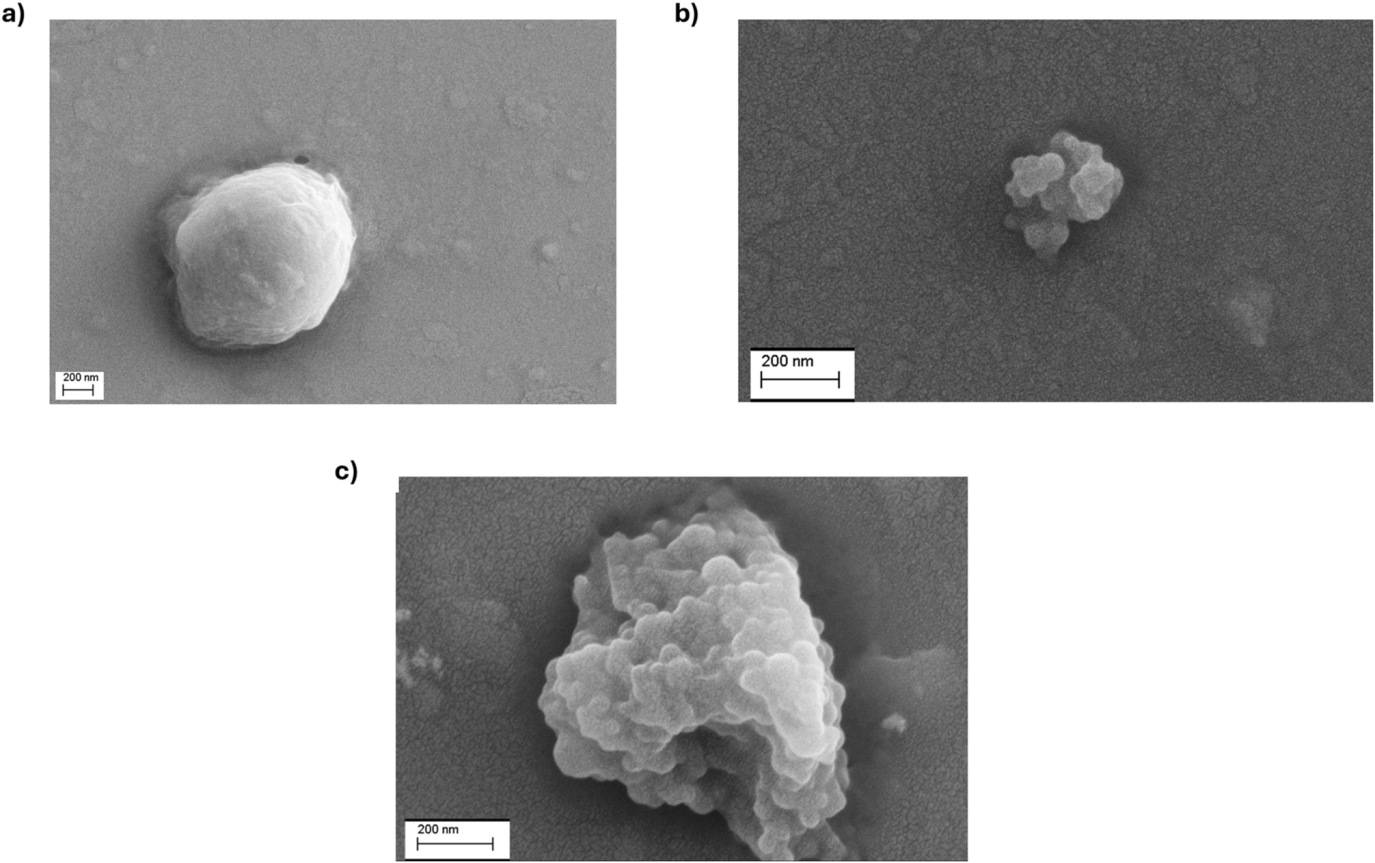
FESEM images of post AF4 fractions of (a) pollen (*R*_t_ 12–18 min); (b) fraction of NanoPET 0.015 mg mL^−1^ (*R*_t_ 10–14 min) and (c) the fraction of the second and third population of MIX2_overnight (*R*_t_ 12–18 min).

NanoPET and pollen suspensions were mixed (see Materials and methods 2.3 in SI) and the resulting samples analyzed by AF4-multidetection platform. The results were compared to injections of the same mass of NanoPET alone (Fig. 5a, black profile) and pollen (Fig. 5a, green profile), serving as references for the “profilomic” approach to better identify the potential interaction. Though the pollen reference was more diluted than previous analyses (Fig. 2a), similar results were obtained, with a size distribution between 220 and 420 nm and an average *R*_g_ of 339 nm (Fig. 5a, green profile), compatible with inhomogeneous solid spheroids reaching 800–1000 nm of diameter. This confirmation shows that pollen is stable in water and its profile is not influenced by dilution. For both reference analyses, we collected fractions downstream AF4 separation for morphological characterization by FESEM. The image of pollen AF4 fraction (Fig. 6a) shows a small micrometric particle (∼1–1.5 μm) with irregular shape attributable to the presence of pollen grains, while no molecular and macromolecular species could be imaged. It is noteworthy the difference in the size between the natural pollen supernatant (∼15 μm) and the fraction of pollen supernatant (∼1–1.5 μm). Larger particles like those found in the non-fractionated undiluted pollen (Fig. 1c) were not visible in the collected fraction of diluted pollen. Probably, larger species are heavier and less abundant, and therefore, given the dilution of the sample, are either statistically more unlikely to be found, or deposited in the injection vial or on the surface of the membrane during the focus step.

FESEM imaging of the AF4 collected NanoPET reference fraction (Fig. 6b) reveals particle aggregates, with individual particles clearly visible and measuring approximately 60–80 nm in diameter. The sizes obtained for both pollen and NanoPET references by FESEM are consistent with the size distributions obtained from MALS, providing cross-validation of the dimensional characterization between the two techniques.

The NanoPET–pollen mixes were injected into the system both immediately and after incubation for 24 h, showing different fractogram profiles (see the example of MIX2 in Fig. 5a). In the fresh samples, no meaningful peaks appear in the time range where NanoPET usually elutes; instead, a shifted single peak is observed, with a size distribution slightly higher than that of the pollen supernatant. Since co-elution of NanoPET and pollen is highly unlikely due to their very different sizes, this suggests that NanoPET may interact with pollen components through surface adsorption and the formation of loosely bound complexes. This limited size increase and the noisy size distribution may reflect a dynamic equilibrium between free and interacting pollen particles, leading to an overall size distribution not distant from that of clean pollen. Additionally, if NanoPET adhered extensively and stably to the pollen surface, a more pronounced increase in the radius of gyration (*R*_g_) would be expected. But we cannot expect by default that there is a complete coating, especially since nanoPET is also involved in the interaction with smaller molecules and other species. Then, it is likely that only a few nanoPET particles are depositing per pollen particle, and a compatible shift in time (hydrodynamic radius) and *R*_g_ is observed. Overall and most importantly, these observations indicate that interactions could occur even after short times.

Interestingly, the mixture samples injected after 24 h showed that new stable conjugates formed in the solution, and the profile changed accordingly. Three peaks appear in the fractogram Fig. 5a (red profile), among which a peak at 8.7 min and a shoulder peak at 13 min appeared for the first time, while the third population has the same retention time as pollen reference, eluting at 16 min. Upon examining the UV absorption spectrum of the peak at 8.7 min (Fig. 5c, red profile), it is apparent that there is a notable abundance of NanoPET nanoparticles. Furthermore, absorption is also detected in the range of 400–500 nm, corresponding to the characteristic absorption band of pollen (Fig. 5c, green profile). So, it is likely that the plastic absorbs some small soluble pollen components and changes polarity, forming an ‘eco-corona’ that causes the movement of a part of the plastic to the upper layer in the channel during the elution process, gaining a higher velocity and being eluted at the beginning of the elution step. This effect means that nanoPET with an ecocorona has higher distance from the membrane (and lower interactions with it), and in fact the peak containing nanoPET in the mix is narrower than that of nanoPET alone. A hypothesis is that the presence of sugars and other molecules lower the absolute value of the PET zeta potential (that is negative), allowing for a softer double layer. The UV/Vis iso-absorbance plot of MIX2_overnight shows clear bands at 250, 270 and 490 nm for the populations eluting at 13 and 16 minutes (Fig. 5b), having the same absorption bands as the first population at 8.7 min (Fig. 5c, red profile). After AF4 processing, two fractions of this sample were collected, corresponding to the first population at 8.7 (Fraction #1) min and the peaks eluting between 12 and 18 min (Fraction #2). The FESEM images of Fraction #2 show many NanoPET particles as micrometric aggregates (Fig. 6c). These aggregates appear larger than those in the NanoPET reference (Fig. 6b), suggesting that NanoPET particles may depose on pollen grains and form hybrid structures.

**Fig. 7:**
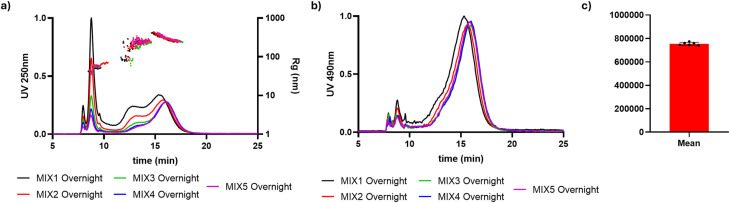
AF4-UV/Vis fractograms of MIX 1–5 overnight at (a) 250 nm and (b) 490 nm. (c) Mean area of fractograms at 490 nm for MIX 1–5 overnight and pollen reference, each black point represents the fractrogram area of one of the listed samples.

To further verify the rationality of the previous analysis, we explored the interaction in mixtures with constant pollen content and various concentrations of NanoPET (Table S3) injected after 24 h of incubation. As shown in Fig. 7a, the absorbance intensity of the peaks at 8.7, 13 and 16 min increases with the concentration of NanoPET. Additionally, the UV/Vis fractogram peak area at 490 nm is constant for all mixtures and pollen reference (Table S5), with a relative standard deviation (RSD) of 1.7% (Fig. 7c). This indicates that pollen components remain in suspension and that the presence of NanoPET does not induce their precipitation but rather leads to interaction.

In fact, the absorption peak at 8.7 min is visible at 250 nm (NanoPET selective absorption) and also at 490 nm, and both signals increase with NanoPET concentration (Fig. 7a/b). Since the size distribution of population at 8.7 min (*R*_g_: 40–70 nm) and UV/Vis characteristic spectrum analysis (Fig. 5c, red profile) shows the presence of NanoPET, we confirm the dose–dependent interaction with soluble pollen components. Indeed, on the one hand, NanoPET retains its size but changes retention time, indicating a change in surface charge and subsequent repulsion from the AF4 membrane, with the loss of the original peak, and, on the other hand, mixtures and pollen reference area at 490 nm is constant but the signal at 8.7 min increases with plastic concentration, indicating that small, dispersed pollen-derived molecules may coat the NanoPET surface. This is a direct detection of the formation of an eco-corona in the native state, in aqueous suspension without chemical modifications, and a crucial finding to evaluate nanoplastics behavior upon dispersion in the environment.

The size distributions of the populations at 13 and 16 minutes are very similar to the one of pollen, but FESEM analysis of the corresponding fractions shows the presence of PET nanoparticles, putting forward the hypothesis of pollen grains surrounded by PET nanoparticles. This is confirmed by the absorption band at 490 nm in the UV/Vis iso-absorbance plot (Fig. 5b) and the representative spectrum of those populations (Fig. 5c, red profile), characteristic of pollen, and the constant peak at 16 min in the fractograms at 490 nm, compatible with the constant amount of pollen added in the mixes. Again, the interaction between NanoPET and matrix is proven and differentially detected, both as NanoPET becoming more stable in suspension and eluting faster, and as NanoPET being carried by bigger species acting as trojan horse.

In conclusion, the coupling of dynamic (AF4) and static (FESEM) analysis highlights the interaction between pollen and NanoPET in mixture samples, suggesting two different interactions: the soluble macromolecules of pollen can cover the NanoPET particles forming of an eco-corona, while the big colloidal species of pollen are covered by the NanoPET.

#### 3.3.2. DEP-Raman analysis

A further approach that we employed in nanoplastics monitoring in support of those previously discussed is the combination of dielectrophoresis and Raman microspectroscopy (DEP-Raman). Given that Raman spectroscopy is typically applied to samples of microscopic dimensions or larger due to its inherent spatial resolution limit, this optical technique is innovative in the field of nanoparticle analysis.^53^ It also suits the aforementioned profilomic approach: since Raman spectra are additive and most synthetic polymers present sharp and intense features in specific spectral regions, the presence of micro- and nanoplastics can be tracked even in small quantities by monitoring deviations from the spectral profiles of uncontaminated samples. Meanwhile, DEP facilitates Raman analysis in many complex liquid matrices — including biological, food, or environmental samples like solute-rich or muddy water, milk, or blood — by manipulating, separating, and/or concentrating particles, often bypassing the need of preprocessing as opposed to typical analytical setups (*e.g.* digestion, filtration, bulk separation). In particular, for this study focussing on the interaction between NanoPET and bee pollen, DEP was employed to separate free NanoPET particles from those interacting with pollen by opting for appropriate electrical parameters, in order to analyze suspended pollen and only those polymeric particles interacting with it.

The DEP-Raman approach was used to chemically characterize the pollen–NanoPET MIX samples summarized in Table S3. With the selected electrical conditions unassociated NanoPET nanoparticles do not accumulate and move away from the confocal volume, but a NanoPET spectrum was acquired as control by changing the voltage bias frequency. NanoPET spectrum is reported in Fig. S3, with all the characteristic PET peaks. For the characterization of the pollen–NanoPET mixtures in this study, we focus on the peaks at 3080 cm^−1^, corresponding to the aromatic C–H bond stretching, at 1726 cm^−1^ to the C=O stretching, and at 1615 cm^−1^ to the C–C of band ring stretching,^54^ as they are distinguishable from the pollen signals because of their strength and wavenumber positions.

**Fig. 8:**
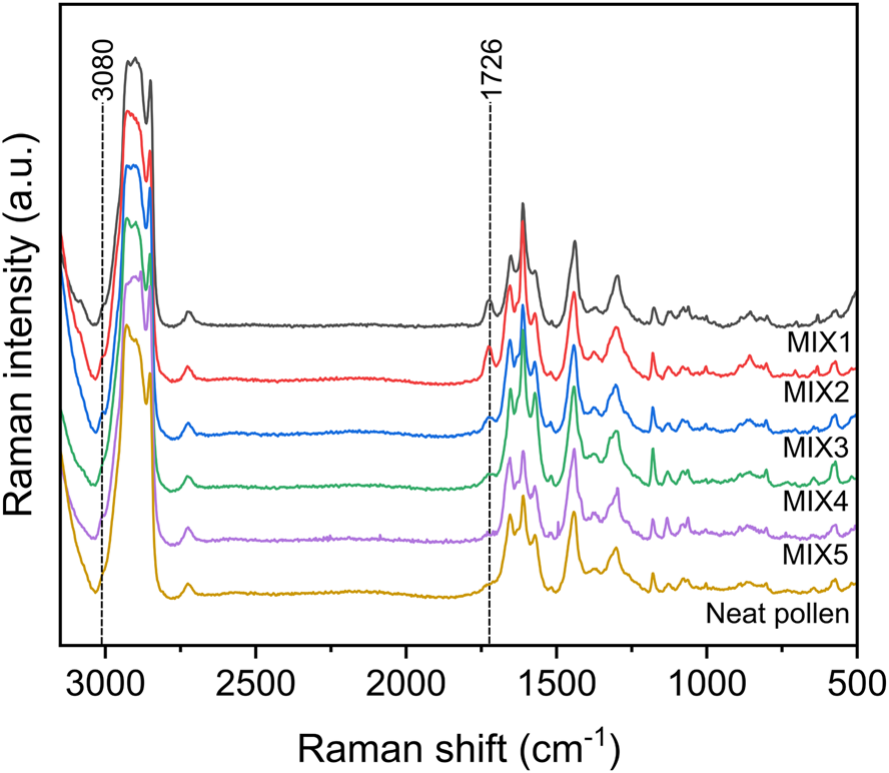
Raman spectra of (from the bottom) neat pollen, MIX5, MIX4, MIX3, MIX2, and MIX1 in the spectral range between 3100 and 500 cm^−1^. Peaks at 3080 cm^−1^ and 1726 cm^−1^, characteristic of NanoPET, are highlighted with a dashed line.

Neat pollen and pollen–NanoPET suspensions (MIX1-5) Raman spectra are shown in Fig. 8. Different from NanoPET, the accumulation time before acquisition for neat pollen suspension was 30 s. All the spectra of pollen–NanoPET suspensions present the characteristic peaks of neat pollen reference and, additionally, peaks at 3080 cm^−1^ and 1726 cm^−1^, characteristics of NanoPET which intensity increases with NanoPET concentration in the mixture. This intensity increase was also observed at 1615 cm^−1^, a band that combines PET and pollen signals. As a conclusion, the Raman spectra indicate that the presence of pollen in the mixture leads to the accumulation of NanoPET adsorbed to the pollen surface.

It must be emphasized that the use of the profilomic approach has, for the first time, allowed the identification of a diagnostic peak through the DEP-Raman technique in a complex matrix, as NanoPET interacting with pollen in aqueous environment.

#### 3.3.3. Py-GC-MS orthogonal validation and coupling feasibility

The successful analysis of NanoPET *via* Py-GC-MS confirmed the possibility to integrate AF4 with analytical pyrolysis for this study. The method involving HFP yielded a satisfactory recovery of 77% ± 12% for NanoPET. Therefore, this procedure was used to chemically characterize the AF4 fractions obtained from the NanoPET–pollen mix.

**Fig. 9:**
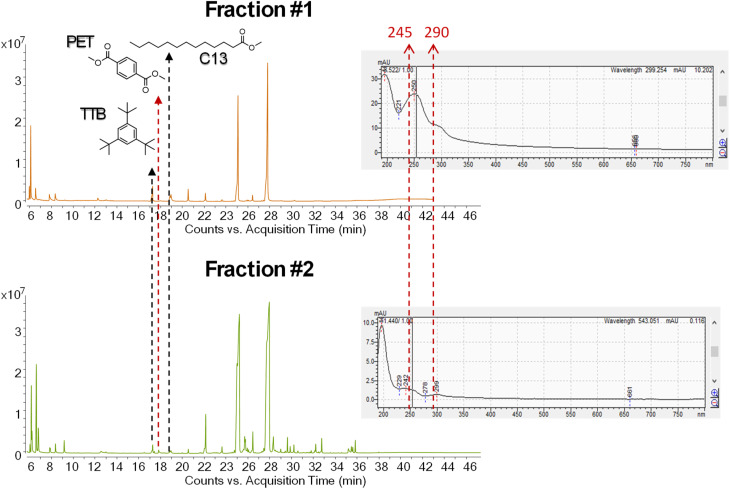
Total Ion Chromatogram (TIC) of two AF4-fractions analyzed by reactive Py-GC-MS with TMAH and respective UV-absorption spectra.

To improve and support the integration of AF4 with Py-GC-MS it was decided to increase the amount of both NanoPET and aqueous pollen injected in the separation system to increase the sensitivity of the method. The separation process resulted in the collection of two fractions (Fraction #1 and #2, Fig. 9) respectively centered at 9 and 15 minutes of the separation method, where the UV-Vis/DAD detector showed the characteristic spectra of PET. Py-GC-MS analyses also confirmed the presence of NanoPET in both fractions. Based on the amount of aqueous pollen injected into the separation system, visible signals of its pyrolysis products were expected in the Total Ion Current (TIC) chromatogram. However, their detection was more challenging than anticipated, suggesting a possible loss of material during the lyophilization step.

In addition, several peaks were found in the pyrograms, many of which originated from the solubilization of contaminants by HFP during sample pre-treatment as confirmed by procedural blanks.

To detect low-intensity signals from pyrolysis products of the pollen, target ion chromatogram extraction was performed. Based on prior thermal characterization, tetra-*O*-methyl-3-deoxy-d-*arabino*-hexanoic acid methyl ester, tetra-*O*-methyl-3-deoxy-d-*xylo*-hexanoic acid methyl ester, 1,2,4-trimethoxybenzene and 1,2,3-trimethoxybenzene were identified as proxies for the presence of pollen. Target ions were selected from the mass spectrum of each compound and the pyrograms of the fractions were extracted accordingly: *m*/*z* 129, 101 for monosaccharide derivatives and *m*/*z* 168, 153, 125 and 110 for trimethoxybenzenes. The simultaneous presence of all the target ions at the expected retention times confirmed the presence of these compounds in Fraction #2 This result indicates the presence of pollen in that fraction, confirming the species interaction *via* an orthogonal method to UV absorption, and highlighting the potential of obtaining advanced results by coupling AF4 and Py-GC-MS for quali-quantitative analyses of NPs and the investigation of chemical species composing different fractions.

## 4 Conclusions

PET nanoplastics, coherent with a real case scenario of pollen sampling from bees, were characterized, using both static techniques and the FFF colloidal approach. We propose a fast and cheap method to characterize the size modification of NanoPET in the presence of water-soluble macromolecular pollen species and we developed and tested a combined methodology that enables nanoplastics profiling, offering insights into fingerprint variations, while also characterizing changes in aggregation state, solubility, and eco-corona formation. Specifically, the successful combination of separation (AF4) and imaging (FESEM) revealed two distinct interaction mechanisms: the formation of a soluble macromolecular corona surrounding NanoPET particles and the coating of pollen grains by NanoPET. The NanoPET–pollen interaction was further confirmed by Py-GC-MS analysis of the AF4 fractions, which identified characteristic ions from both components, and by DEP-Raman spectroscopy on the unprocessed mixtures, where signals from both pollen and NanoPET were detected after the separation of unreacted nanoparticles. The “profilomic” approach combined with the AF4 technique, along with innovative benchmarks and direct mass measurement (Py-GC-MS) confirmations, provided fast, cheap, and green information on pollen–NanoPET interactions by avoiding the use of burdensome pretreatment protocols, such as enzymatic digestions. Moreover the results highlight the value of interpreting Raman data within a multidimensional analytical framework that provides a broader context of particle behavior and environmental interactions, thereby enabling a more comprehensive and informed spectral interpretation. Overall, our innovative approach allowed for clear identification and continuous monitoring of NanoPET plastics, as well as tracking their transformation into more stable forms through coating with pollen-derived components. The proposed “profilomic” approach stands out for its high analytical sensitivity, throughput, and sustainability, as it enables rapid and informative screening of complex environmental interactions without exploiting targeted analytical techniques. These features promote applicability in environmental monitoring, offering a valuable contribution to the investigation of NPs behavior and their biological impact, *e.g.* including their influence on pollen aggregation and buoyancy, which may affect pollination or other ecological processes.

The formation of the eco-corona on PET nanoparticles may influence the environmental distribution and deposition patterns of both pollen and nanoplastics. These ecological impacts demand further investigation, as understanding the dynamics of particle settling and transport could provide important information for future research on assessing and managing the environmental risks associated with nanoplastic pollution.

Additionally, the presence of other organic matter in the environment may further modulate PET–pollen interactions, introducing additional complexity to the system. Understanding how different components influence the behavior of plastic nanoparticles could provide crucial insights not only into their environmental fate, but also into how the formation of the eco-corona might be induced and controlled. This knowledge may support the development of mitigation strategies aimed at reducing the mobility, bioavailability, or toxicity of nanoplastics in ecological systems.

## Conflicts of interest

There are no conflicts to declare.

## Supplementary Material

RA-015-D5RA03254G-s001

## Data Availability

The data supporting this article have been shown in the main manuscript and included as part of the SI. Sample properties and method development, and reference spectra. See DOI: https://doi.org/10.1039/d5ra03254g.
