# Supplementary material for: Multitechnique characterization of eco-corona formation on airborne nanoplastics
Source: RSC Adv. 2025 Aug 28;15(37):30849–64. doi: 10.1039/d5ra03254g (PMC12395032; doi:10.1039/d5ra03254g)
Supplement: RA-015-D5RA03254G-s001 [file RA-015-D5RA03254G-s001.pdf]

Supplementary Information (SI) for RSC Advances.
This journal is © The Royal Society of Chemistry 2025

Multitechnique Characterization of Eco-Corona Formation on Airborne Nanoplastics

Anna Placci[1], Marta Fadda[2], Irene Coralli[3], Junjie Wang[1], Andrea Zattoni[1,4], Anna Luisa Costa[5], Raquel Portela[6], Andrea Mario Giovannozzi[2], Daniele Fabbri[3], Dora Melucci[1], Stefano Giordani[1], Barbara Roda[1,4], Pierluigi Reschiglian[1,4], Simona Ortelli*[,5], Alessio Sacco*[,2], Valentina Marassi*[,1,4]

1. Department of Chemistry "G. Ciamician", University of Bologna, Via Piero Gobetti 83, 40129 Bologna, Italy

2. Quantum Metrology and Nanotechnology Department, National Institute of Metrological Research (INRiM), Strada delle Cacce 91, 10135 Torino, Italy

3. Department of Chemistry "Giacomo Ciamician", Technopole of Rimini, University of Bologna, Via Dario Campana 71, 47922 Rimini, Italy

4. byFlow s.r.l, Via dell'Arcoveggio 74, 40129 Bologna, Italy

5. CNR-ISSMC, National Research Council of Italy - Institute of Science, Technology and Sustainability for Ceramics,Via Granarolo 64, 48018 Faenza (RA), Italy

6. CSIC - Instituto de Catalisis y Petroleoquimica (ICP),, C/ Marie Curie, 2. 28049 Madrid, Spain

Supplementary information

Sample name	Provider	Concentration (mg/ml)	Size from DLS (nm)	Dispersant	PdI	ζ-POT (mV) ; pH	Shape
NanoPET	CSIC	4.9	97.5	water	0.5	-31 ± 1; 7	spherical

Table S1. NanoPET sample properties

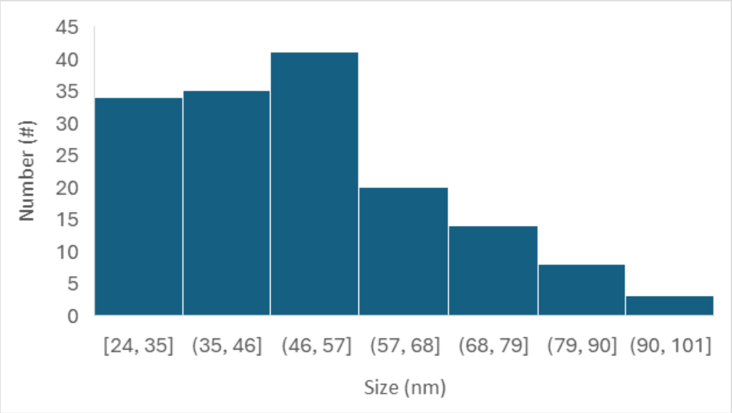

Figure S1. NanoPET particle size distribution obtained by FESEM image analysis on more than 150 particles.

Standards name	Provider	Concentration (% m/w)	Mean hydrodynamic diameter (nm)	Dispersant	Shape
PS_50 nm	Duke scientific corporations	1	50 ± 2	water	spherical
PS_200 nm	Duke scientific corporations	1	204 ± 6	water	spherical
PS_300 nm	Duke scientific corporations	1	300 ± 5	Water	spherical

Table S2. Polystyrene standards properties

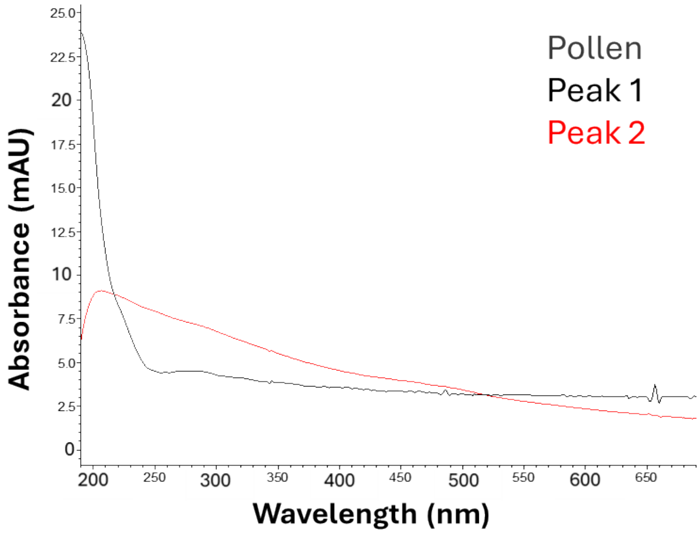

Figure S2. Absorbance spectrum of Peak 1 (Retention time: 8 min) and Peak 2 (Retention time: 16 min) of pollen supernatant analyzed through AF4 multidetection platform

Mixture sample	Pollen dilution factor	NanoPET concentration (mg/ml)
MIX1	4	0.0300
MIX2	4	0.0150
MIX3	4	0.0075
MIX4	4	0.0038
MIX5	4	0.0019

Table S3. Plastic and pollen mixtures preparation. Reference samples in Table S4

Detector flow (mL/min)	0.5
Injection flow (mL/min)	0.5
Injection time (min)	7.0
Cross flow (mL/min)	2.0 (0-9 min)<br>2.0-0.01 (9-49 min, exp. 0.1)<br>0.01 (49-59 min)<br>0.0 (59-64 min)

Table S4. AF4 separation method parameters used in the AF200 instrument

AF4 method development

During AF4 method development, optimization of the duration and the flow rates (focus flow and cross flow) in each step typically revolves around achieving good separation and recovery (1). For our instrument, usually the initial cross flow rate directly determines the focus flow rate during the focus step. The initial cross flow was set as a median of 2 ml/min, according to previous work experience and plastic particle studies (2, 3), for 7 min while the sample was injected in the channel. To support the complete particles release from the accumulation wall, another 2 min constant cross flow of 2 ml/min was set at the beginning of elution step.

The decay mode of cross flow is another critical factor affecting the separation resolution. Three different decay mode with exponential value 0.1, 0.2, and 0.25 were chosen for testing, in which the exponent 0.1 showed the best separation resolution for the bigger particles. Considering our plastic and pollen samples characteristics, the exponent 0.1 was chosen for the next measurements. An elution time of 40 min allows all the species to elute out from the channel.

Using the optimized parameters, 50 nm, 200 nm, and 300 nm PS standard samples eluted at different retention times (Rt): 50 nm (Rt 10.9 min), 200 nm (Rt 14.4 min), and 300 nm (Rt 17.3 min); this means that satisfactory size separation of the PS nanoparticles was achieved.

Sample	P-REF	MIX 1	MIX 2	MIX 3	MIX 4	MIX 5	STDEV	RSD
Peak area (490 nm)	739279	773393	745408	745373	758891	759380	12594.02	1.7 %

Table S5. Fractogram at 490 nm areas for Pollen reference (P-REF, pollen diluted 1:4 in water) and MIXES

DEP-Raman

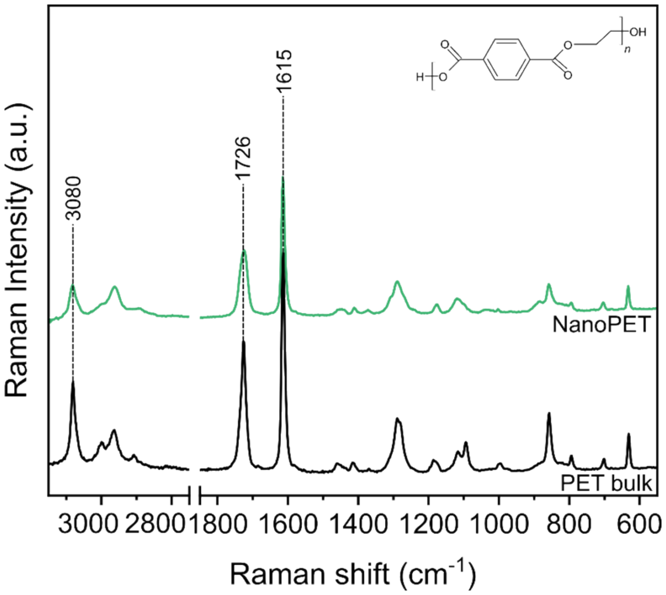

Figure S3. Raman spectrum of NanoPET (green) compared with a macro-PET plastic debris as control with all the assignments relevant for this study.

References

1. Gigault J, Pettibone JM, Schmitt C, Hackley VA. Rational strategy for characterization of nanoscale particles by asymmetric-flow field flow fractionation: a tutorial. Analytica chimica acta. 2014;809:9-24.
2. Schwaferts C, Sogne V, Welz R, Meier F, Klein T, Niessner R, et al. Nanoplastic analysis by online coupling of Raman microscopy and field-flow fractionation enabled by optical tweezers. Analytical chemistry. 2020;92(8):5813-20.
3. Huber MJ, Ivleva NP, Booth AM, Beer I, Bianchi I, Drexel R, et al. Physicochemical characterization and quantification of nanoplastics: applicability, limitations and complementarity of batch and fractionation methods. Analytical and bioanalytical chemistry. 2023;415(15):3007-31.
